# Supplementary material for: Hidden chaos factors inducing random walks which reduce hospital operative efficiency
Source: PLoS One. 2022 Jan 27;17(1):e0262815. doi: 10.1371/journal.pone.0262815 (PMC8794195; doi:10.1371/journal.pone.0262815)
Supplement: Attachment — Submitted filename: Reply to reviewers comments.docx [file pone.0262815.s002.docx]

**Journal Requirements.**

When submitting your revision, we need you to address these additional requirements.

1. Please ensure that your manuscript meets PLoS ONE's style r ….. .

**Reply: We did our best to comply. We used a published PLoS article to reproduce its form information fields an and style. Not all the features were available in tha LaTeX remplate, but we hope that any shortcomings will be easily solved for your typesetters. Also, most journal use and require authos supplied Key Words, we added a small section with keywords to the manuscript, if this is unsuitable it will be trivial for your typesetters to comment or erase the Key Words section which is all in one line.**

2. Has the statistical analysis been performed appropriately and rigorously? Reviewer #1: Yes

Reviewer #2: Yes

**Reply: No reply seems necessary.**

3. Please update your submission to use the PLoS LaTeX template. … ,

**Reply: Done as suggested. This implied a long time modifying each of the entries of our database to conform to PloS ONE uses on doi numbers and url links. It took a long time since the BibTeX dbase contains 3315 entries and it most conform to the ABS book format we use for a book citif 847 references, thus far.It was a lot of work. Perhaps the most important of our references (Sevcik, 1998) appeared in an on line journal , Complexity International, which is no longer available. Ten years ago we deposited a copy of Sevcik 1998 in arXiv, in a manner that complies with the extinct Complexity International Journal copyrights. The link to the arXiv file MUST be on the citation of this work, thus we had to tweak the citation to make the arXiv ling visible even using PLoS One bibliography style.**

4. Thank you for stating the following in the Acknowledgments Section of your manuscript:

**Reply: The information was set in the LaTeX template. It was divided into sections as done, for example in** [**https://doi.org/10.1371/journal.pone.0258052**](https://doi.org/10.1371/journal.pone.0258052) **which we use as styling example.**

5. Please review your reference list to ensure that it is complete and correct. …

**Reply: Our references are stored in an extense BibTeX formatted data base used in all our publications. However, we have to modify the fields thr references in our BiBTrX dbase to accommodate to PloS One bibliography style without destroying the references format for other uses.**

Reviewer's Responses to Questions

Comments to the Author

1. Is the manuscript technically sound, and do the data support the conclusions?

The manuscript must describe a technically sound piece of scientific research with data that supports the conclusions. Experiments must have been conducted rigorously, with appropriate controls, replication, and sample sizes. The conclusions must be drawn appropriately based on the data presented.

Reviewer #1: Yes

Reviewer #2: Yes

**Reply: No reply seems necessary.**

2. Has the statistical analysis been performed appropriately and rigorously? Reviewer #1: Yes

Reviewer #2: Yes

**Reply: No reply seems necessary.**

3. Have the authors made all data underlying the findings in their manuscript fully available?

**Reply: We are unsure about the meaning of the YES choice by the reviewerss. It could mean in the context of the question that we did it right in the manuscript, or that we should include more. In any case we are including two MS Exel-formatted files with the original La Fuenfría Hospital data. The manuscript, we feel, it is clear on how was the data used and corrected for a short breech of missing data. In any event, we are open to any questions from the readers. The original HLF spreadsheets provided have Spanish labels and headings, but each of these may be easily translated to any language using Google Translate^®^ for example.**

Reviewer #1: Yes

Reviewer #2: Yes

4. Is the manuscript presented in an intelligible fashion and written in standard English?

**Reply: As pointed out by Reviewer 2, there were some typos in the paper. We revised once more, but since our capacity to detect mistakes in our papers decreases as we re-read them, we hired a professional proofreader. We hope that now the issues are fixed.**

Reviewer #1: Yes

Reviewer #2: No

5. Review Comments to the Author.

Please use the space provided to explain your answers to the questions above.

**Reviewer #1:**

The paper is very interesting and well structured. The analysis conducted is appropriate and well communicated. The implications of the findings are also clearly outlined. However, the paper needs proof reading due to typos or missing punctuation; e.g. page 8 line 18 - These shows; page 8 line 20 and page 7 line 7 missing commas; page 9 line 40 - ''such as described'' should read ''such as those described..''

**Reply: Thanks for your kind opinion. The revised version will be proof read by a certified professional English proofreader. Prior to this, we re-read the manuscript and corrected the typos we were able to see.**

**Reviewer #2:**

The aim of the authors is to show that factors not usually considered in hospital performance analysis may have a huge impact on hospital efficacy - by delving into the case of La Fuenfría Hospital. The methodology is explained in a way that enables replication and the discussion and conclusions triangulate with the rest of the paper. However, the paper has many typos and grammatical mistakes, which makes it a difficult read and sometimes understand. Even the abstract seems like a literal translation from the original language and does not flow well. I suggest an complete proof read and edit of the article by a native English speaking person.

6. PLoS authors have the option to publish the peer review history of their article ([what does this mean?](https://journals.plos.org/plosone/s/editorial-and-peer-review-process#loc-peer-review-history)). If published, this will include your full peer review and any attached files.

If you choose “no”, your identity will remain anonymous but your review may still be made public.

**Do you want your identity to be public for this peer review?** For information about this choice, including consent withdrawal, please see our [Privacy Policy](https://www.plos.org/privacy-policy).

**Reply: We appreciate Prof. Grima openness.**

Reviewer #1: No

Reviewer #2: **Yes:** Prof. Simon Grima
